# Cancer testis antigen expression in triple negative breast cancer: Candidate targets for cancer immunotherapy?

**DOI:** 10.1186/2051-1426-3-S2-P381

**Published:** 2015-11-04

**Authors:** Wouter Hendrickx, Mariam Al-Muftah, Julie Decock

**Affiliations:** 1 Sidra Medical and Research Institute, Doha, Qatar; 2 Qatar Biomedical Research Institute, Doha, Qatar

## Background

Breast cancer is a major health concern in Qatar with a younger age at diagnosis and projections of 60% increase in new cases. Triple negative breast cancer (TNBC) is associated with advanced disease at diagnosis and poorer outcome, and can be subclassified into 6 gene-expression-based subtypes. These patients don't benefit from endocrine or HER2-targeted therapy and represent 15-20% of cases mandating the need for novel treatments. Although immunotherapy has shown promising results in different cancers, there are only 2 clinical trials to date assessing adoptive cell immunotherapy in TNBC. Cancer testis antigens (CTA) could be good candidate targets as their expression is often up-regulated in malignant tissues, while it is restricted in the testis and absent or very low in other somatic tissues.

## Methods

We mined the TCGA and NCBI GEO repositories for genomic data on CTA expression in TNBC and selected a panel of 15 CTAs for further investigation. Gene and protein expression was investigated in a series of 9 human TNBC cell lines, encompassing all subtypes.

## Results

We found the gene expression of TSAG10, MAGEA5, PLAC1, and DKKL1 to be moderate/highly expressed in our cell lines and in both datasets, and are confirming this on protein level. We are establishing a biobank of DNA and RNA of Qatari breast cancer patients and will present gene expression data of CTAs in TNBC tumors.

## Conclusions

Our preliminary findings suggest that TSAG10, MAGEA5, PLAC1 and DKKL1 could be good candidate targets for TNBC immunotherapy, and in particular could benefit Qatari breast cancer patients.

